# Genome-Wide Analysis of PDZ Domain Binding Reveals Inherent Functional Overlap within the PDZ Interaction Network

**DOI:** 10.1371/journal.pone.0016047

**Published:** 2011-01-24

**Authors:** Aartjan J. W. te Velthuis, Philippe A. Sakalis, Donald A. Fowler, Christoph P. Bagowski

**Affiliations:** 1 Department of Medical Microbiology, Leiden University Medical Center, Leiden, The Netherlands; 2 Department of Bionanoscience, Delft University of Technology, Delft, The Netherlands; 3 Institute of Biology, Leiden University, Leiden, The Netherlands; 4 Institute of Pharmacy and Biotechnology, German University Cairo, New Cairo City, Egypt; John Craig Venter Institute, United States

## Abstract

Binding selectivity and cross-reactivity within one of the largest and most abundant interaction domain families, the PDZ family, has long been enigmatic. The complete human PDZ domain complement (the PDZome) consists of 267 domains and we applied here a Bayesian selectivity model to predict hundreds of human PDZ domain interactions, using target sequences of 22,997 non-redundant proteins. Subsequent analysis of these binding scores shows that PDZs can be divided into two genome-wide clusters that coincide well with the division between canonical class 1 and 2 PDZs. Within the class 1 PDZs we observed binding overlap at unprecedented levels, mediated by two residues at positions 1 and 5 of the second α-helix of the binding pocket. Eight PDZ domains were subsequently selected for experimental binding studies and to verify the basics of our predictions. Overall, the PDZ domain class 1 cross-reactivity identified here implies that auxiliary mechanisms must be in place to overcome this inherent functional overlap and to minimize cross-selectivity within the living cell. Indeed, when we superimpose PDZ domain binding affinities with gene ontologies, network topology data and the domain position within a PDZ superfamily protein, functional overlap is minimized and PDZ domains position optimally in the binding space. We therefore propose that PDZ domain selectivity is achieved through cellular context rather than inherent binding specificity.

## Introduction

Protein-protein interactions play key roles in many cellular processes and are mediated by specific interaction modules called domains [1], [2]. These domains are conserved, functionally autonomous protein sequences, which can behave as independent genetic elements within genomes [3] and bind to ligands containing a core structural motif. Currently, 1777 domain superfamilies are recognized in the latest release of the Structural Classification of Proteins (SCOP) [4], each with its own abundance per genome and tendency to recombine [5].

**Figure 1:**
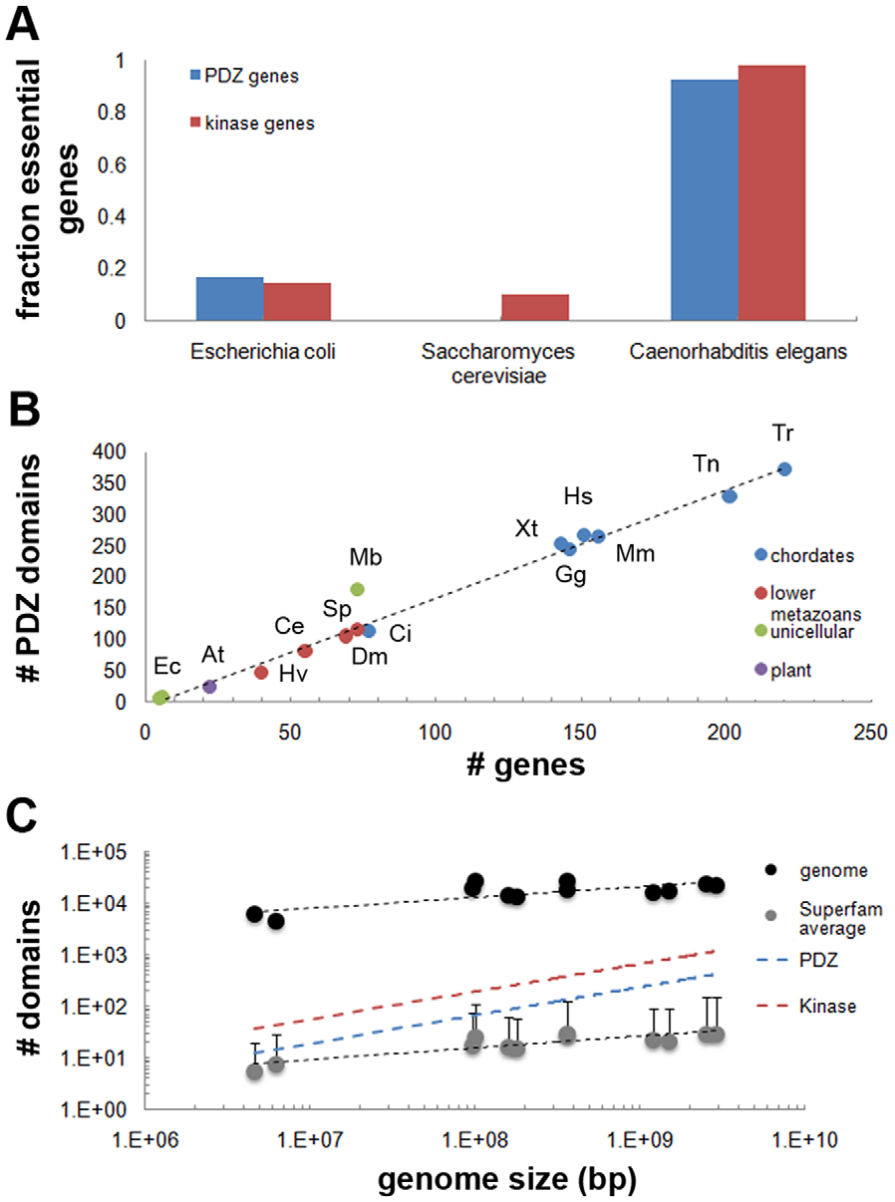
Overview of PDZ domain complements (PDZomes) encoded in model organisms. (**A**) The fraction of essential PDZ domain encoding genes rises with increasing genome size. Essential genes were scored for *Caenorhabditis elegans* (92.7% of all PDZ encoding genes), *Escherichia coli* (16.7%) and *Saccharomyces cerevisiae* (0%). See also file S1. (**B**) Plot depicting a linear relation between the number of PDZ domains and the number of genes that encode them. The mean ratio of PDZs per gene was 1.7 and relatively constant among metazoan organisms. Interestingly, the PDZ to gene ratio was higher for the unicellular choanoflagellate *Monosiga brevicollis* (Mb), and this unicellular species encoded more PDZ domains and PDZ encoding genes than both lower metazoans and bacteria [9], [21]. (**C**) Logarithmic plot of genome size and domain content shows that the PDZome increase can be approximated by a power-law (blue). A similar result was observed for the kinase domain superfamily (red). Error bars for the average superfamily size indicate standard deviation.

Among protein interaction domains, PDZ domains are one of the most abundant domains encoded in the human genome. Their importance is emphasized by the fact that over 20 heritable human diseases have been attributed to malfunctioning PDZ containing proteins or their ligands [6], [7]. Furthermore, our assessment of the percentage of lethal genes within the PDZ domain complement (termed here ‘PDZome’) showed that 92.7% of all PDZ encoding genes are essential in the nematode *Caenorhabditis elegans* (Fig. 1A, file S1).

Canonical PDZs typically fold to modular structures composed of 80–100 amino acid residues and bind short C-terminal sequences [8] in an elegant mechanism that allows interaction without disrupting ligand structure and function [9]. However, in spite of extensive studies aimed at understanding the biochemical and structural basis of the PDZ inner-workings, PDZs have proven difficult to place into discrete functional categories [10]. Currently, PDZs are widely regarded as relatively promiscuous interaction domains, which may have specificity for more than one target protein provided that the target is of a certain ligand class. An example is the fifth PDZ domain of mouse protein Magi3, which was shown to bind at least 25 different ligands in a recent peptide library study [11].

Using the specificity of PDZ domains for certain ligands, a scheme of classes was formulated [8]. Currently, the best recognized classes are class 1 PDZs, which bind c-terminal motifs with the sequence X-S/T-X-Φ-COOH; class 2 PDZs, which have micromolar affinity for the sequence X-Φ-X-Φ-COOH; and class 3 PDZs, for ligands which end with X-D/E X-Φ-COOH. In this scheme, X represents any amino acid residue and Φ any hydrophobic residue.

Many questions have nevertheless been raised concerning the restrictiveness of the canonical classes of PDZ domains and the presence of more alternative specificity clusters [12], [13]. Two groundbreaking studies aimed at addressing this controversy found very limited binding overlap and proposed that PDZs are evenly distributed across the ligand specificity space [10], [14]. Indeed, the wide range of ligand binding variations was therefore sophisticatedly visualized by Tonikian et al [14] with position weight matrices (PWMs) instead of the canonical scheme. However, both studies did not discuss their findings in relation to the numerous domain and gene duplications that are also a hallmark of the PDZ superfamily [15], [16], and thereby allowed room for speculation about the existence of potentially significant functional overlap among PDZ domains due to their similar, and occasionally even identical, binding pockets. PDZ selectivity, which we for simplicity define here as the final and reproducible outcome of target binding in the cell in the presence of other potential ligands, may thus still be relatively weak on the genomic level, particularly given the already extensive protein-domain promiscuity and the micromolar specificity of the domains described to date. However, no clear estimations are presently available on the scale of the human genome.

To gain insight into the binding of PDZ domains on this genomic scale and the actual impact of sequence similarity on the functional overlap of PDZ binding, we firstly identified the PDZ domain complement of the human genome and fourteen other genomes. Subsequently, using the Bayesian selectivity model formulated by Chen et al that was based on a large set of PDZs of various organisms [17], we calculated the most likely interactions of 96% of all PDZ domains encoded in the human genome. Subsequent analysis, including site-directed mutagenesis studies, showed that two specificity clusters exist in each of the investigated PDZomes, and that the presence of a histidine and valine residue in alpha-helix 2 (residues αB1 and αB5, respectively) determines the binding of the most promiscuous cluster. These observations are in accordance with previously published data on the presence of class 1 and 2 PDZs [8] and class 1 PDZ binding [18], [19]. Our analysis additionally revealed an extensive functional overlap within class 1 PDZ binding pockets. This apparent lack of inherent selectivity questions the functionality of the PDZ binding space if it were the only parameter involved in organizing PDZ-protein interactions. We therefore speculate here how optimization and specificity can be achieved by taking into account the protein interaction network that encompasses the PDZ interactions in a living cell.

## Results

### The PDZome and its expansion

In order to obtain the most comprehensive picture of PDZ binding selection, we assembled the PDZomes of 15 species through Hidden Markov Model identifications [20], iterative comparison of several vertebrate and invertebrate genomes and cross-comparison of seven major databases. Our analysis revealed that the human PDZome is dispersed over 152 genes, encoding a total of 267 PDZ domains (Fig. 1B, S1B, S2, File S2). This is the third highest PDZ domain per gene ratio (1.8∶1) of all 15 organisms investigated and these numbers also indicate that various previous estimates which relied on single databases were either under- or over-estimations. The highest ratio was found for the protozoan *Monosiga brevicollis* (2.5∶1), which also encodes significantly more PDZ domains than plants and lower metazoans (Fig. 1B). This unicellular organism thus appears to be an exception to the hypothesis that PDZ domains co-evolved with multicellularity [9], [21] and it differs from the evolutionary more distant unicellular organisms such as *Dictyostelium* and *Tetrahymena* that do not bear coding regions for PDZ domains [22]. Interestingly in this regard is the identification of a second type of PDZ in the structural analysis of the D1p protein [23] and biochemical analysis of RseP [24]. This PDZ shares little sequence identity with canonical PDZs as a result of a circular permutation, which significantly effects the PDZ's folding pattern [25] and explains why it was not identified in genomic analyses and may potentially forebode the identification of PDZs of the same type in other unicellular organisms. Currently, however, little is known about its binding mechanism.

Our dataset furthermore shows that the PDZome size (and the number of genes encoding a PDZ domain) correlates well with genome size by a power-law (Fig. 1C), an important aspect of important modular domains [26] (see also file S3). Adding to the overall importance of the PDZ domain, we found that the expansion of the number of PDZ domains, i.e. the PDZ superfamily size, showed a steeper increase than the mean expansion of all superfamilies (Fig. 1C, Fig. S2 and file S3).

### PDZ binding by PDZomes

**Figure 2:**
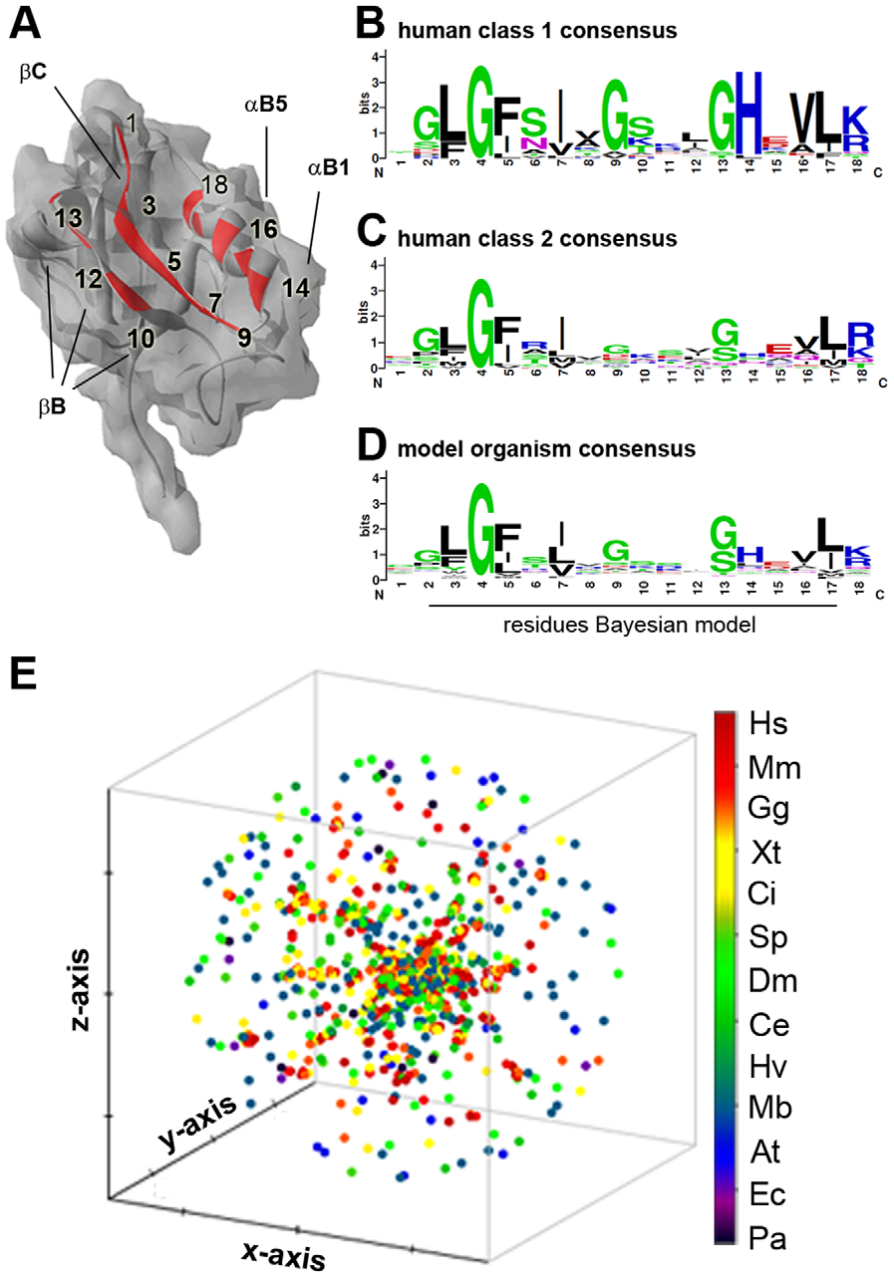
The PDZ binding pocket. (**A**) PDZ binding pocket residues were identified through molecular modeling and sequence alignment (see file S4). We extracted the 16 residues and two flanking residues, previously identified to be of importance for ligand binding [14], [17]. (**B**) Consensus sequence of the amino acids in the binding pocket of class 1 PDZs as defined by Tonikian et al. [14] (n = 22). (**C**) Consensus sequence of human class 2 PDZs (n = 17). (**D**) Consensus sequence of the distribution of amino acids per position of all 15 species investigated in this study. No significant difference was found with the *Homo sapiens* consensus sequence. (**E**) Pair-wise clustering in 3D space of all 1704 PDZ binding pocket sequences identified illustrates specific clustering of metazoan PDZ sequences (red, yellow and green) and independent clustering of the majority of unicellular sequences (green). For a 2D image please see Fig. S3.

As mentioned above, PDZ domains are promiscuous and their specificity is generally confined to one of two ligand classes, although intermediates have been described [15], [16]. Taking into account the considerable contribution of PDZ domains to the proteome, this raises questions about their selectivity and a functional overlap in the cell. Canonically, PDZs fold to modular structures that bind C-terminal ligands via 16 key binding pocket residues, which are dispersed over alpha-helix 2 and beta-sheets 2 and 3 (βB and βC) [10], [14], [17] (Fig. 2A). Most of these residues are not involved in the dynamic responses that take place upon ligand binding. Detailed analysis of the sequences of the PDZ classes proposed recently [14] illustrates that each class has its own binding pocket consensus (Fig. 2B and C), which suggests that the primary PDZ structure can be used to predict PDZ binding. Indeed, several studies have attempted to capture the binding mechanism of PDZs in a selectivity model, using approaches based on Domain Interaction Footprints (DIF) [27], specificity maps [14], dissociation constants [28], trigram and bigram frequencies [29], support vector machines (SVMs) [30], and Bayesian models [17]. However, only the models of Chen et al [17] and Hui and Bader [30] were trained on PDZ domain binding data obtained from multiple species (including human, worm and fly PDZ domains), making them thus the best choice for a multi-species analysis.

In order to identify the PDZ binding pockets of the human PDZome, we combined molecular modeling with multiple sequence alignment (MSA). After multiple rounds of iteration and manual correction, we were able to obtain binding pockets for 254 human PDZ domains (96% of the total 267) at the default Swiss-Model confidence level [31]. A similar approach was performed for the PDZ binding pockets of 14 other model organisms studied here. The overall binding pocket consensus is shown in Fig. 2D (see Fig. 2E and S3 for cluster analysis, and file S4 for alignment). No significant differences were found among vertebrates or between vertebrates and invertebrates (data not shown), suggesting an even and a conserved distribution of PDZ classes among the species investigated.

**Figure 3:**
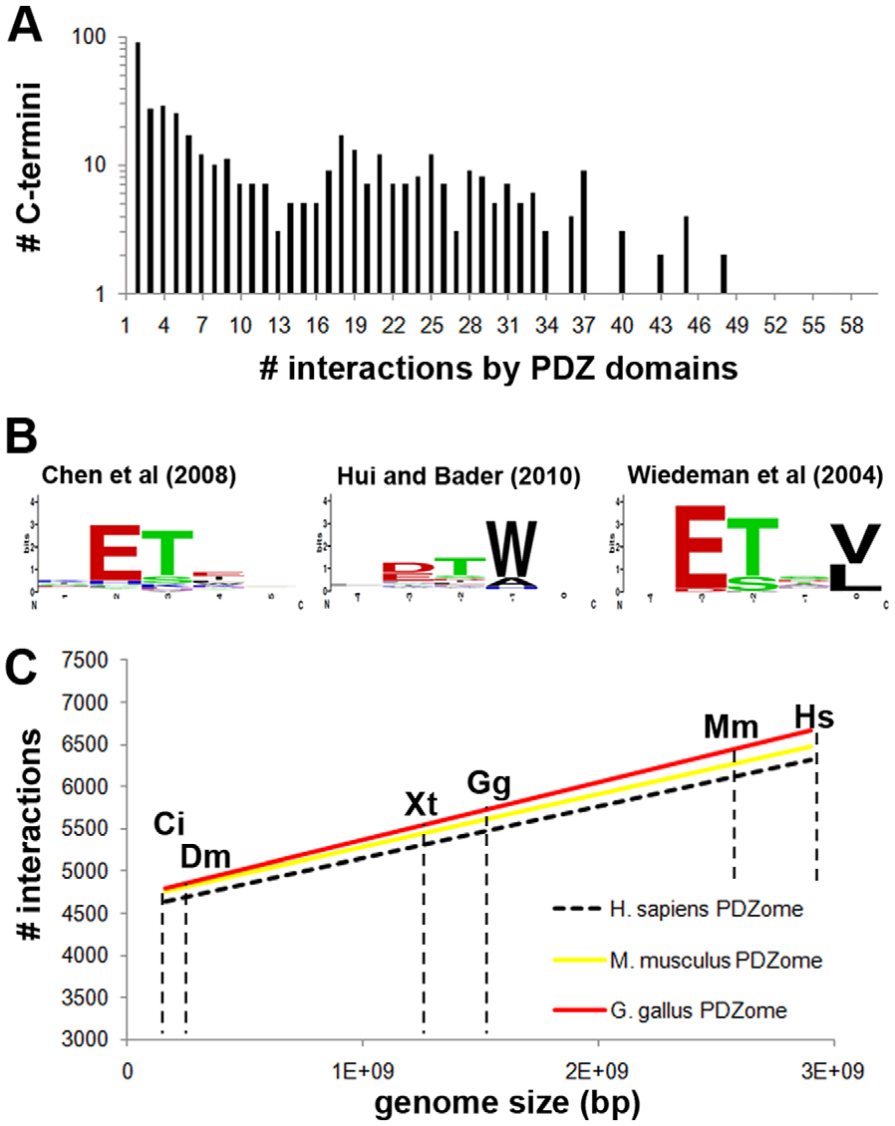
Extensive binding overlap within the PDZome. (**A**) Implementing a Bayesian model for PDZ domain binding prediction, we used 254 PDZ domain binding pockets (96% coverage of the human PDZome) identified to predict the number of PDZ domain interactions with 22,997 human C-termini that were derived from Ensembl longest transcript sequences. The mean ± s.e.m number of PDZ ligands per domain was 24±3.7, which strongly suggested that the PDZ domain binding pockets are not inherently optimized to minimize cross-selectivity within the human cell. (**B**) Comparison between PMWs computed using the human C-termini and the binding pockets of Erbin using the predictive models of Chen et al [17], Hui and Bader [30], and Wiedeman et al [47], for which we used a cut-off of Kd<100 µM. (**C**) Using the PDZ binding pockets identified for other vertebrates in this study, we also found extensive overlap in these species. Cross-comparison of the binding capabilities using six sets of C-termini (proteomes used are marked by dashed lines and a two letter species abbreviation) reveals that for the three vertebrate PDZomes investigated no significant differences were observed as assessed by student t-test (P>0.8). All numbers were normalized to the *Homo sapiens* PDZome.

To obtain quantitative insight into the number of C-termini a human PDZ can bind within a proteome, we applied the Bayesian model of Chen et al [17] to a set of 22,997 human protein sequences (Ensembl longest transcripts) and filtered all interactions predicted to have a binding score above a threshold of 0.798. This threshold is defined as the cut-off that predicts whether a PDZ will bind a given ligand with Kd<100 µM with a false positive rate (FPR) of 6.27% [17]. Using this procedure, we found that 65 PDZs were predicted to bind similar C-terminal ligands (a total of 436) with a mean ± s.e.m. (standard error of the mean) of 24±3.7, implying that these PDZ domains extensively cross-react within the human proteome (Fig. 3A). Moreover, in this genome-wide prediction analysis we mapped a total of 6161 PDZ domain-based interactions, mediated through only 254 PDZ domains.

To qualitatively validate this result we analyzed published experimental data of 123 human PDZ interactions (PDZbase) and found that this set perfectly corroborated that human PDZ domains are not evenly distributed throughout their binding space, which is auxiliary evidence for their promiscuity (Fig. S4). In addition we used the SVM method of Hui and Bader [30] as an independent algorithm to assess our genome-wide predictions. Similarly to the PDZbase data, we found extensive binding overlap between the PDZs in terms of raw numbers although with a distribution that was mildly shifted towards even more binding overlap between PDZs (Fig. S5). Since prediction methods may potentially favor some interactions over others due to the type or coverage of the training set used, a characteristic that may be masked by the number of interactions, we also confirmed that the PWMs of the two genome-wide interaction predictions were relatively similar. Overall we found good agreement between the two models (file S5), as is exemplified by the PWMs obtained for the well studied PDZ domain of Erbin (Fig. 3B).

Furthermore, using five additional C-termini datasets, we performed a cross-PDZome binding analysis for the three vertebrate species in this study (*Homo sapiens*, *Mus musculus* and *Gallus gallus*), and normalized these findings to the human PDZome. This analysis revealed quantitatively that no significant differences exist between the vertebrate PDZomes investigated (P>0.8) and that the inherent functional overlap within the PDZ superfamily is conserved among vertebrates (Fig. 3C).

### The PDZome binding scores can be clustered in two major classes based on class 1 ligands

**Figure 4:**
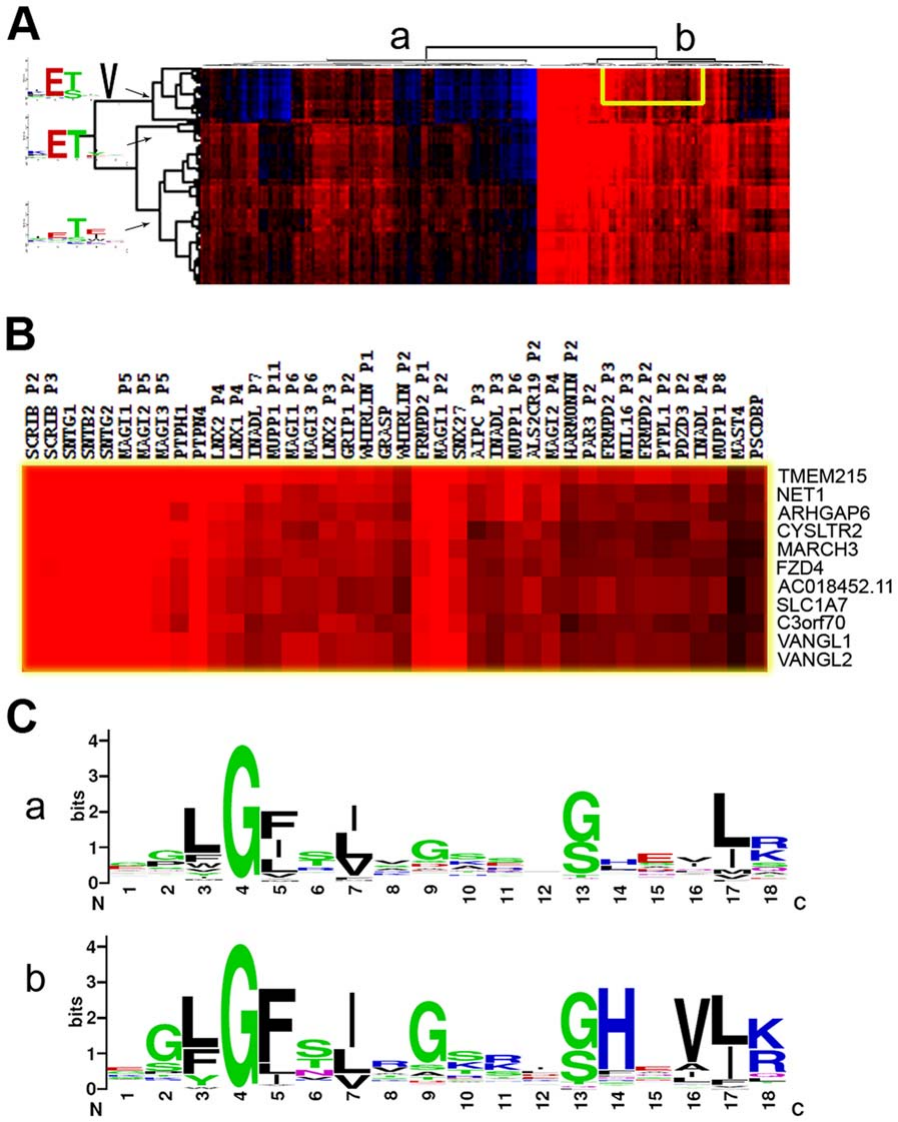
Cluster analysis reveals two amino acids that determine promiscuity. (**A**) Genome-wide hierarchical clustering was used to place human C-termini and PDZ domains with similar binding profiles in close proximity. This image shows a heat map for three identified ligand clusters (for a full image see Fig. S6) and reveals two main PDZ groups: a ligand specific group (marked ‘a’) with on average 1 ligand and a promiscuous group (marked ‘b’) with on average 55 ligands, both at a FPR of 6.27%. Positive psi scores are indicated in red and the negative scores are indicated in blue. Ligands are presented by their amino acid consensus. (**B**) Close-up of the yellow box in Fig. 1A. (**C**) Binding pocket sequence analysis of the two PDZ clusters reveals the preference of a histidine residue at position αB1 (80%) and a valine at position αB5 (70%) in promiscuous PDZ domains (group b), whereas no preference was found in the non-promiscuous PDZ domains.

Through genome-wide hierarchical clustering of the PDZ binding scores we identified two main PDZ groups: a ligand-specific group with on average 1 ligand (147 PDZs) and a promiscuous group with on average 55 ligands (107 PDZs) (Fig. 4A, S6). Interestingly, all interactions proposed by the Bayesian model of Chen et al were mediated through class 1 ligands and of the consensus sequence X-S/T-X-Φ-COOH (Fig. S5). This finding was supported by analysis of the binding pocket consensus of the promiscuous PDZ cluster, which revealed the preference of a histidine residue at position 14 (αB1, present in 80% of the binding pockets) and a valine at position 16 (αB5, present in 70% of the binding pockets; Fig. 4C panel b). This histidine at position αB1 is a major determinant of class 1 PDZs as it forms hydrogen bonds with the T/S residues of the ligand and is thus crucial for binding [18]. Both residues were not found to be overrepresented in the non-promiscuous group (Fig. 4C panel a). Identical results were obtained for the cluster analysis of the two other vertebrate species' binding pockets (*Mus musculus* and *Gallus gallus*).

**Figure 5:**
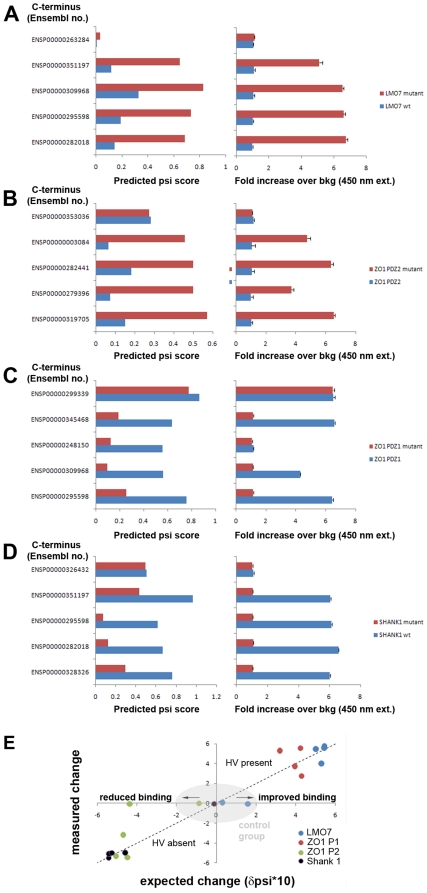
Mutational analysis of PDZ promiscuity. Mutational analysis of human LMO7 PDZ (**A**), ZO1 PDZ 2 (**B**), ZO1 PDZ2 (**C**) and SHANK PDZ (**D**) binding through the mutation of positions αB1 and αB5 of the binding pocket conferred binding or induced a loss of binding to the C-terminal peptide sequences indicated with Ensembl numbers. Use of a negative control peptide (as predicted by the Bayesian model) did not show increased binding. Error bars represent SD (n = 6). (**E**) An overview of mutational analysis of human PDZ domains shows that the mutation of positions αB1 and αB5 to histidine and valine residues increased binding to peptides selected by the binding model (file S7), whereas their mutation to other residues resulted in loss of binding (see file S6 and main text for mutation details, and file S8 for raw data).

To confirm that the interaction modeling had provided testable results and that the residues indentified in our cluster analysis indeed were able to contribute to class 1 PDZ binding promiscuity, we performed a mutational study in which we either mutated αB1 and αB5 of non-promiscuous PDZs to respectively a histidine and valine, or mutated the histidine and valine in PDZs that were computed to be promiscuous to residues found in comparable binding pockets of non-promiscuous PDZs (see file S6 for details). The increase or decrease in binding affinity was then experimentally determined for a set of C-terminal ligands encoded in the human genome that were, according to the Bayesian model, either very weak or strong ligands. In total, we tested 8 different PDZ domains (see file S6) in our binding experiments with each 5 different ligands (see file S7). As shown in Fig. 5A, the mutation of the serine and glutamic acid to a histidine and valine residue at position αB1 and αB5 of the human LMO7 binding pocket conferred binding for ligands that were not bound by the wild-type PDZ domain. A similar result was observed for the introduction of these two key residues into the binding pocket of ZO1 instead of the leucine and lysine present in the wild type ZO1 PDZ (Fig. 5B). In contrast, a loss of binding was observed for ZO1 PDZ2 and the PDZ domain of SHANK1 when the histidine and valine residues were mutated to leucine and lysine or serine and glutamic acid, respectively (Fig. 5C–D). The mutational study thus validated the Bayesian selectivity model well and gave support to the predictions. Additionally, the presence of a histidine and valine at the positions αB1 and αB5 appears critical for class1 ligand binding (Fig. 5E), which is in agreement with studies of the class 1 PDZ of Erbin [14], [18] (for raw data please see file S8).

Taken together, these findings strongly suggest that the human PDZome is not inherently optimized to prevent cross-selectivity of class 1 PDZs and they argue against conclusions drawn from previous studies of mouse [10] and human PDZs [14]. Clearly, a more detailed and structured model is needed to explain how the cell resolves the issue of functional overlap and how an efficient cellular PDZ interaction network is crafted.

### A hypothesis for optimization of PDZ binding selectivity through cellular context

Several studies have demonstrated that protein-protein interaction mapping can be used to help understand complex cellular processes, in which proteins are defined by nodes and positioned in a network through connections based on experimental or mathematical evidence [32]. Moreover, since proteins often contain multiple interaction domains, it is the sum of these interactions that defines the protein's position in the network. In addition, interaction networks can be separated in gene ontology (GO) components to visualize a proteins functional role in the cell.

**Figure 6:**
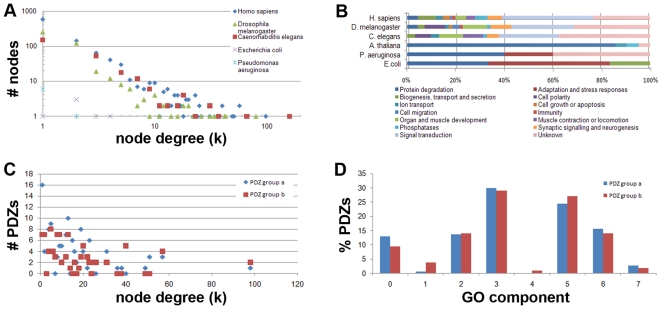
PDZ protein interaction networks. (**A**) Degree analysis of PDZ interactomes shows that all three metazoan networks tested are best approximated by a power-law. (**B**) Metazoan PDZ encoding genes are largely involved in signaling processes, whereas limited divergence in GO functionality was observed for the PDZ genes of plants and bacteria, for which we mainly categorized protease functions. (**C**) Comparison of the ligand specific group of PDZ domains (a) to the promiscuous PDZ domain group (b) (described in Fig. 2) shows that both are evenly distributed over the GO components. Numbers refer to: 0: unknown, 1: nucleus specific, 2: cytoplasm specific, 3: plasma membrane specific, 4: mitochondria specific, 5: multiple compartments but non-nuclear, 6: all compartments, 7: extracellular. (**D**) Distribution by node degree of the two PDZ promiscuity clusters as described in Fig. 4. There was no significant difference found between the two distributions in C (P>0.30) and D (P>0.90), as assessed by student t-test.

To study whether the network neighborhood, in absence of intrinsic selectivity optimization of PDZ domains, could explain the biological mechanisms used to position human PDZs optimally in the cellular space and avoiding binding overlap, we extracted interaction data from four different databases (MIPS, AtPID, BIND and HPRD) and analyzed their consensus in VisANT, while at the same time superimposing the dataset with GO annotations. Our analysis of the connectivity of all PDZ containing proteins in the human protein network revealed a scale-free behavior (Fig. 6A). We found that GOs were distributed among 11 important biological processes (Fig. 6B) and did not observe any significant difference between the distribution of promiscuous PDZ domains and non-promiscuous PDZ domains over the high degree nodes in the network (Fig. 6C) or over the GO compartments (Fig. 6D). This even distribution of the promiscuous PDZ domains over the cellular compartments and interaction neighborhood implies that the average promiscuous PDZ complement is reduced locally in the cell, which in turn suggests that the binding overlap and the cross-reactivity are also resolved locally within the cellular context.

**Figure 7:**
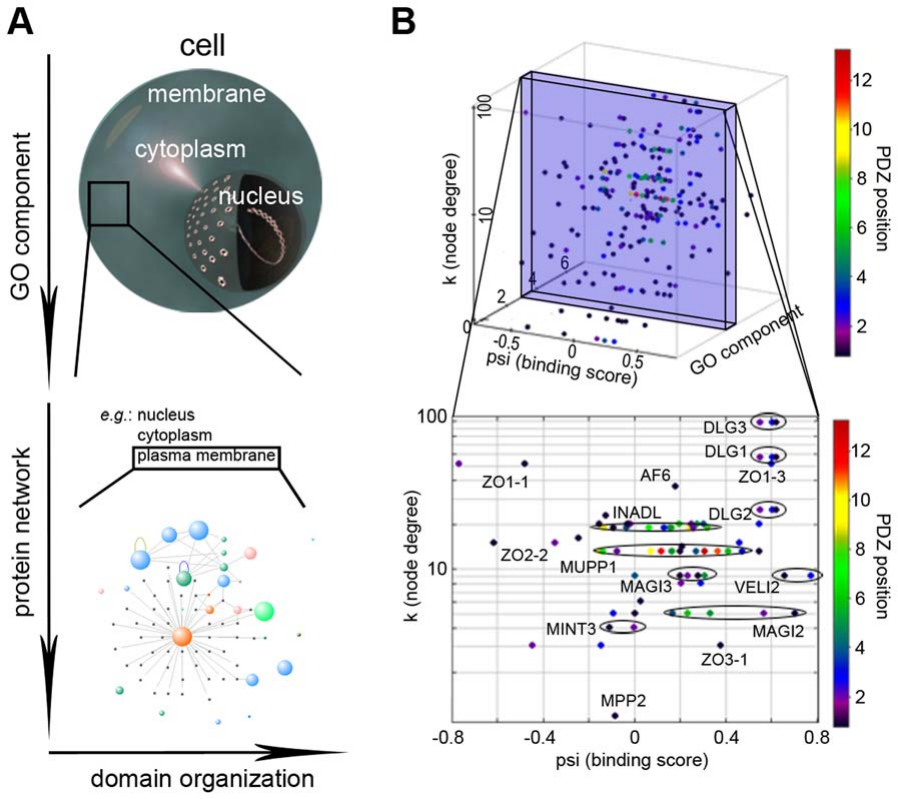
Optimization of PDZ binding selectivity in a four dimensional space. (**A**) Schematic illustration of the stepwise mechanism that reduces inherent PDZ binding overlap in the living cell. (**B**) Practical illustration of the proposed mechanism through interactions with the human plasma membrane protein Neurexin-1. Modeled binding scores are subdivided in cellular space by superimposing gene ontology compartment annotations (numbers as in Fig. 3) and the PDZ protein's node degree. The PDZ position in the protein is color-coded to indicate the presence (e.g. INADL) or absence (e.g. ZO1) of neighboring PDZ domains with similar binding score for (i.e. chance to bind) a given ligand. In the lower panel, PDZ domains of the same protein are encircled to illustrate this. In the cell, having PDZ domains with overlapping binding affinities in a single protein may bring about competition for a specific ligand, but may also increase the overall potential of a PDZ protein to bind the right ligand. Alternatively, splice variations can be envisioned to further specify PDZ selectivity *in vivo*.

The suggestion for the presence of intrinsic binding overlap within the PDZome requires a new model to explain how the cell resolves the issue of cross-reactivity and how a functional PDZ interaction network is crafted. Superimposing consensus network categorical data in a four-step process, hence plotting PDZ binding affinity, gene ontology, node degree and domain positioning in the protein, the number of potential PDZ interactions for a highly interacting protein is greatly reduced and inherent cross-selectivity largely resolved (Fig. 7). This figure also nicely demonstrates how related (i.e., duplicated) PDZs of the same protein fall within the same binding affinity range (Fig. 7).

## Discussion

Protein-protein interactions are mediated by specific interaction domains, which are relatively conserved, functionally independent protein sequences that behave as independent genetic elements within genomes and can bind to specific ligand classes. Regarding the PDZ domain, however, the latter issue has remained largely enigmatic due to conflicting findings concerning the restrictiveness of the canonical classes of PDZ domains, their physiological affinity range, their binding selectivity (or rather ‘binding promiscuity’) and the size of the PDZ family.

Through careful cross-examination of multiple databases, we established here that the human PDZome contains 267 PDZ domains, distributed over 152 genes and that the PDZome in general has expanded more quickly over the metazoan genomes than the average superfamily size. A correct determination of the PDZome size and the binding sequences important for the interactions mediated by PDZ domains is crucial as domain and gene duplications are wide-spread across the genome and are particularly manifested among PDZ encoding gene families [15], [16]. An underestimation of the PDZ content and the number of duplications with high sequence conservation (some PDZ proteins encode 13 relatively closely related PDZ domains [33]) would lead to an incorrect sampling of the PDZ binding space. Furthermore, certain PDZ domains have been found to be more promiscuous towards their targets than others and exclusion of these from the dataset would introduce a significant second bias.

Using the Bayesian modeling procedure published by Chen et al., we found a total of 6161 PDZ domain based interactions, mediated through only 254 PDZ domains. These data suggest that multiple PDZs can bind similar C-terminal ligands with a Kd<100 µM, in turn implying that these PDZ domains extensively cross-react within the human proteome (Fig. 3A). Consistent with the scale of the findings presented here, a recent classification study indentified 3100 interactions between a set of random ligands and 88 human PDZ domains [14]. Nevertheless and even though the model was trained on PDZ domains of various organisms, and appears to work well for the set of PDZ domains tested here, we have to mindful of the fact that a fraction of the interactions may not have been correctly predicted or missed.

Recently, it was hypothesized that this may, in part, be a fundamental aspect contributing to the dynamic regulation of post-synaptic density (PSD) signaling complexes, since the presence of multiple ‘slots’ prevents disengaged receptors from outward diffusion, whereas a more densely packed PSD would be less mobile [34]. At the same time, the relatively weak PDZ interactions ensure that modifications to the PSD organization can be triggered rapidly [35]. On the other hand, further resolution of PDZ-dependent binding and complex association may be achieved through phosphorylation of a particular PDZ domain [36], or via phosphorylation of the C-termini of PDZ targets [37], [38], through the protein complexes themselves, as they can hide potential binding partners in their inner core [34], or potentially through the expression of splice variants lacking one or more domains. Taken together with the speculative model mentioned above (Fig. 7), the data thus brings forward a new hypothesis for binding overlap reduction inside the cell that can be further investigated in the future.

In summary, we have modeled here an extensive set of PDZ-C-terminal ligand interactions encoded in the human genome, which can be used to guide future cell biology experiments. We find on a genome-wide scale that binding promiscuity of class 1 PDZ domains is regulated by two key amino acid residues at position αB1 and αB5 of the binding pocket (Fig. 2A) and that their genome-wide presence confers significant cross-reactivity to the PDZome and class 1 ligands in the PDZ-based protein interaction network. However, the distribution of promiscuous class 1 PDZs across the interactome and cellular compartments is even and we therefore postulate that the human PDZ binding selectivity is optimized through its cellular context in order to resolve the biological problem of intrinsic cross-selectivity. Since the genomes encode high numbers of other promiscuous interaction domains, we suggest that their binding selectivity may be regulated in a similar multidimensional way.

## Materials and Methods

### PDZ dataset

A database PDZ domains was created using the databases from Ensembl (http://www.ensembl.org/index.html), National Centre for Biotechnology Information (NCBI, http://www.ncbi.nlm.nih.gov/), pFam (http://www.sanger.ac.uk/Software/Pfam/), Superfamily version 1.68 (http://supfam.mrc-lmb.cam.ac.uk/Superfamily/), UniProt Knowledgebase (http://www.expasy.org/sprot/), SMART (http://smart.embl-heidelberg.de/smart/) and the TranSignal PDZ Domain Arrays (http://www.panomics.com). PDZ sequences obtained from these databases were aligned with MUSLCE 3.6 to filter redundant entries. Cross-species comparisons were subsequently performed and suspected redundant or missing entries blasted against genome sequence and EST databases (Ensembl, Tigr, NCBI) to identify overlapping or missing gene loci, respectively. Newly identified sequences were examined for correct intron-exon sites, reliable alignment with homologs and for the presence of HMMER identifiable domains. The PDZome dataset was ultimately established for the following species: *Homo sapiens* (Hs), *Mus musculus* (Mm), *Gallus gallus* (Gg), *Xenopus tropicalis* (Xt), *Tetraodon nigroviridis* (Tn), *Takifugu rubripes* (Tr), *Ciona intestinalis* (Ci), *Drosophila melanogaster* (Dm), *Caenorhabditis elegans* (Ce), *Hydra vulgaris* (Hv), *Strongylocentrotus purpuratus* (Sp), *Monosiga brevicollis* (Mb), *Arabidopsis thaliana* (At), *Escherichia coli* (Ec) and *Pseudomonas auruginosa* (Pa). All sequences retrieved and their associated annotations can be viewed in file S2.

### PDZ binding matrix

The previously established scoring matrix for PDZ C-terminal sequence binding [17] relies on both the identification of 16 key amino acid residues in the structurally conserved binding pocket of the PDZ domain and 5 residues of the C-terminal ligand. To implement this Bayesian matrix we identified these 16 residues through MSAs made with MUSCLE version 3.6 [39], and structural models that were created with the SWISS-MODEL workspace (E-value limit 1.0E-6) and refined and visualized with Swiss-PdbViewer version 3.7 [31]. From the refined alignments, we extracted 18 residues (including the two flanking residues) and applied these to the LabView 8.6-based algorithm of the Bayesian interaction model proposed by Chen et al. [17] (file S9, with lookup table, the human PDZ binding pockets and human C-termini provided as file S10, file S11 and file S12 respectively; separate files for binding pockets of all other species can be obtained from the authors upon request). Interactions according to the SVM model were calculated using the data from Hui and Bader [30].

A dendrogram of the identified PDZ binding pockets was created with the Neighbor-Joining algorithm from the Phylip package (version 3.6) [40] and visualized with HyperTree [41], whereas 3D clustering was performed in Clans version 4 [42], based on PSI-Blast pair-wise alignment scores generated with the Blossom-80 matrix.

To calculate and predict PDZ interactions for a non-redundant set of human c-terminal sequences, the Ensembl longest transcript set of protein sequences was downloaded from the Superfamily website (version 1.69) and the c-terminal sequences extracted with a custom LabView algorithm. Clustering of binding scores was performed with Cluster 3.0 and the heat map created with TreeView 1.6. The amino acid distributions for the ligand clusters and the PDZ binding pockets were created with weblogo (http://weblogo.berkeley.edu/logo.cgi).

### Cloning of GST-Fusion proteins and site-directed mutagenesis

All constructs were created as described previously. Briefly, human multi-tissue cDNA (MTC) (Clontech 636747) was used for domain-specific PCRs except for ZO1-PDZ1 and ZO1-PDZ2, for which we used an Image clone as PCR template (BC111712 MGC:133289 IMAGE:40037646). PCR products were cloned in pGEX5 vectors (GE Healthcare) and subsequently used for site-directed mutagenesis with the QuikChange Site-Directed Mutagenesis Kit (Stratagene) according to the manufacturer's protocol. All mutations were confirmed by sequencing.

### PDZ domain expression and purification

pGEX5 vectors harboring PDZ cDNA sequences were expressed in *E. coli* BL21(DE3) using auto-induction medium ZYM-5052 [43]. Bacteria were lyzed in PBS by sonication and the PDZ domain fusion proteins purified with amylose resin (New England Biolabs) according to the manufacturer's instructions. The isolated PDZ domain fusion proteins were analyzed on SDS-PAGE and Coomassie staining according to standard procedures and subsequently aliquoted and stored in 20 mM Tris (pH 7.5), 200mM NaCl, 2mM DTT and 10% glycerol at −20°C.

### PDZ domain binding assay

Biotinylated peptides were designed according to Stifler et al. [44] with an N-terminal tri-peptide (NNG) to increase solubility. 96-well plates were coated with a rabbit anti-GST polyclonal antibody (Sigma) to bind the GST/PDZ domain fusion proteins (4 µg/ml). For the binding assay, biotinylated peptides (4 µM) (purchased from United Peptide Corporation, UPC) were used, which were detected with HRP-Streptavidin and HRP substrate 3,3,5,5-Tetramethylbenzidine (TMB) (Sigma). Reactions were stopped with 1M H_3_PO_4_ and analyzed at 450 nm.

### Interactome and gene ontology analysis

Taking NCBI and Uniprot accession numbers, gene identifiers and synonyms (see file S2) we queried the Gene Ontology database [45] for associated ontology slim terms. Where available, species specific databases were queried for additional information. Data for interactome construction was obtained from MIPS, AtPID, BIND and HPRD. Visualization and analysis of this set was performed in VisANT 3.0 using default settings. To identify whether the genes identified for the PDZome dataset were essential for life of the particular organism investigated, we queried WormBase version 194, the Yeast Deletion Project and the *E. coli* phenotype dataset, assembled by Gerdes *et al.* [46] for (embryonic) lethal mutants.

## Supporting Information

Figure S1 **Comparison of protein domain databases.** (A) Cross comparison of several on line protein domain databases shows large disparities for essentially all species investigated. Often this was found to be due to large amounts of redundant sequences or absence of data from ESTs or partial gene prediction hits. After manual curation we assembled the PDZome database, which contains the best complete and non-redundant set of PDZ domains for the species listed to date. (B) Graph depicting both the number of PDZ domains and PDZ encoding genes found in the various genomes investigated. (C) Logarithmic plot of a subset of species from the PDZome dataset (Hs, Mm, Gg, Xt, Tr, Tn, Ci, Dm, Ce, At, Pa and Ec). This figure also shows the R-square and trend function obtained from the data. (D) Logarithmic plot for the data retrieved from the Superfamily database (http://supfam.mrc-lmb.cam.ac.uk/SUPERFAMILY/), using the similar subset of species as in Fig. S1C. (D) Logarithmic plot for the Pfam (http://pfam.sanger.ac.uk/) and SMART (http://smart.embl-heidelberg.de/smart/set_mode.cgi?NORMAL=1) databases as in Fig. S1C. (E) Logarithmic plot using the full set of species investigated for the PDZome dataset, as in Fig. 1C of the main text. The R-square value of this graph is inferior to the one in S1C, likely as a result of the inclusion of data from unfinished genome projects. (F) Distribution of the number of PDZs per gene per organism in percentages shows that PDZ domains are not evenly distributed over the genes and that multi-PDZ genes are underrepresented. The graph shows furthermore an increase in PDZ gene complexity during metazoan evolution and highly complex genes in *Monosiga brevicollis*, with up to 22 PDZs per gene. The gene complexity in the non-metazoan species investigated is low.
(TIF)

Figure S2 **Relation between domain content and genome.** (A) Correlation between the number of PDZs per genome and the number of PDZ encoding genes. Arrow indicates the *Monosiga brevicollis* data point. (B) Correlation between the number of kinase domains per genome and the number of kinase domain encoding genes. (C) Correlation between the number of SH3 domains per genome and the number of SH3 encoding genes. (D) Correlation between the number of chromo domains per genome and the number of chromo domain encoding genes.
(TIF)

Figure S3 **Clustering of PDZ sequences.** Hierarchical clustering after multiple sequence alignment was color coded for chordate (blue), invertebrate (red) and unicellular (green) species. This illustrates that specific clusters of PDZ domains exist that are specific for unicellular or metazoan species (indicated with brackets). The latter are mostly composed of PDZ binding pocket sequences encoded by the *Monosiga brevicollis* genome, suggesting that these arose specifically in this species and that the *Monosiga brevicollis* PDZ domains were not transferred through horizontal gene transfer, as was proposed previously for unicellular organisms.
(TIF)

Figure S4 **Extensive PDZ binding overlap in other organisms.** (A) Mouse PDZ-ligand interaction predicted by Stiffler *et al.* for a redundant set of mouse proteins. (B) The observation that multiple human PDZ domains bind multiple ligands is also apparent from our analysis of an experiment-based set of interactions extracted from the PDZbase. (C) Number of interactions per PDZ as predicted with the set of 22,997 human C-termini from non-redundant (longest transcript) Ensembl protein sequences.
(TIF)

Figure S5 **Genome-wide prediction of PDZ binding using an alternative algorithm.** Extensive PDZ binding overlap in the human genome as predicted using the method from Hui and Bader. Compared to the results obtained with the method by Chen et al (Fig. 3A), a lot more overlap is seen, with many more C-termini being bound by different PDZs.
(TIF)

Figure S6 **Clustering of PDZ domains and their ligands.** Genome-wide hierarchical clustering was used to place human C-termini and PDZ domains with similar binding profiles in close proximity. Beside clusters of ligands on the left of the cluster graph (presented by their amino acid consensus), this heat map also reveals two main PDZ groups: a ligand specific group (marked ‘a’) with on average 1 ligand and a promiscuous group (marked ‘b’) with on average 55 ligands, both at a FPR of 6.27%. Positive psi scores are indicated in red and the negative scores are indicated in blue.
(TIF)

File S1 **Table showing number of essential genes encoding PDZ, SH3, Kinase or Chromo domains.**
(DOC)

File S2 **Excel file containing the details of the PDZome described in this study.**
(XLS)

File S3 **Supplemental text with more details on the PDZ dataset described in this study.**
(DOC)

File S4 **Sequence alignment of PDZ binding pockets.**
(PS) Click here for additional data file.

File S5 **Table showing a PMW comparison of the two computational methods used in this study.**
(DOC)

File S6 **Table listing the amino acid sequences of the PDZ domains and their mutants that were used for binding analysis.**
(DOC)

File S7 **Table listing the peptide sequences that were used in the binding analysis.**
(DOC)

File S8 **Raw interaction data between PDZ and peptide ligands.**
(DOC)

File S9 **Labview 8.6 code that was used in this study for genome-wide analysis of PDZ interactions.**
(VI) Click here for additional data file.

File S10 **Lookup table required for running the Labview 8.6 code in File S9.**
(TXT)

File S11 **Tab-delimited file containing the Homo sapient PDZ binding pockets that were used for genome-wide prediction of PDZ interactions using File S9.**
(TXT)

File S12 **Tab-delimited file containing the Homo sapiens c-terminal proteins sequences that were used for genome-wide prediction of PDZ interactions using File S9.**
(TXT)
