# Supplementary figures and images for: Genome-Wide Analysis of PDZ Domain Binding Reveals Inherent Functional Overlap within the PDZ Interaction Network
Source: PLoS One. 2011 Jan 24;6(1):e16047. doi: 10.1371/journal.pone.0016047 (PMC3026046; doi:10.1371/journal.pone.0016047)

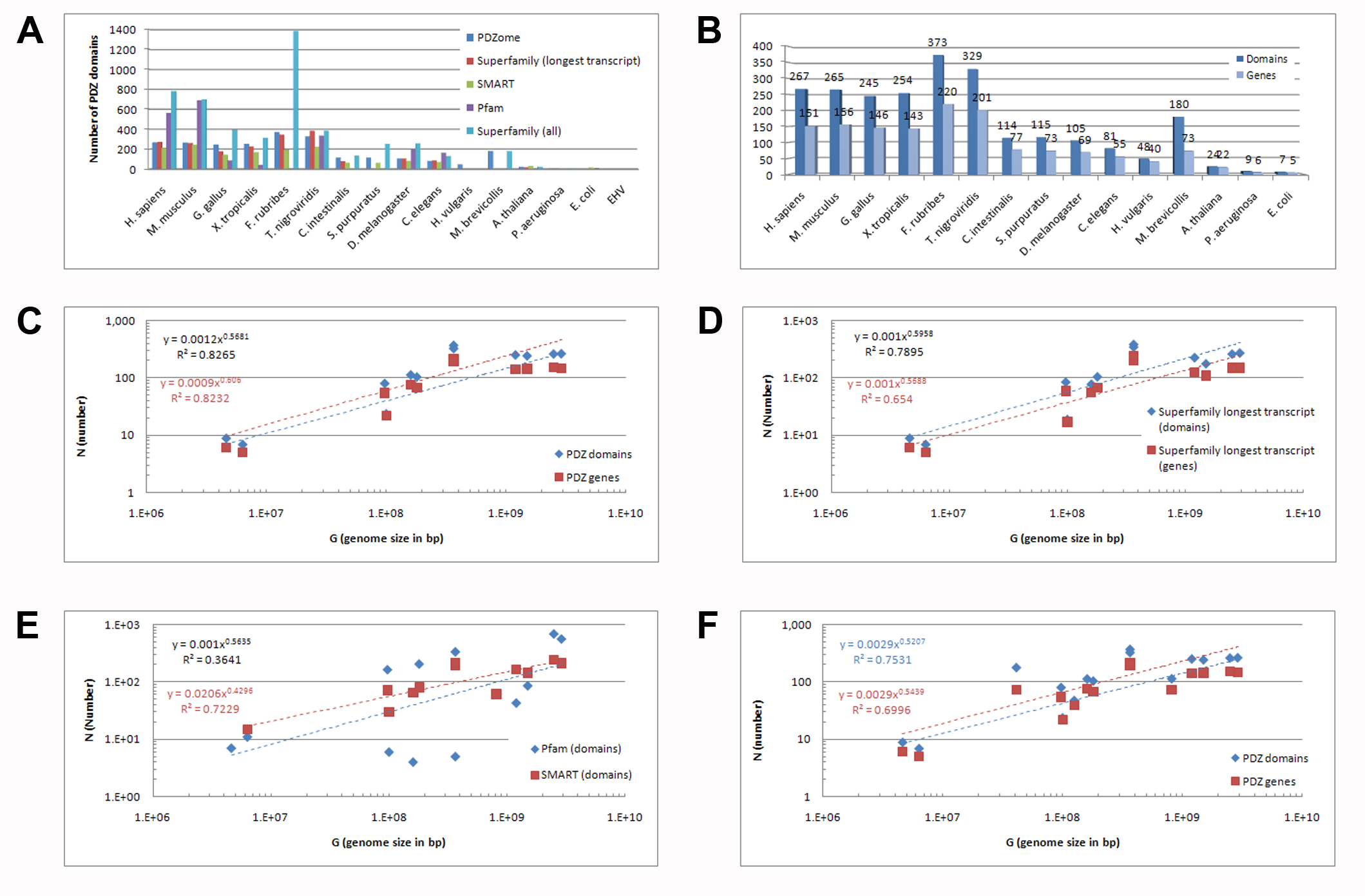

Supplement: Figure S1 — Comparison of protein domain databases. (A) Cross comparison of several on line protein domain databases shows large disparities for essentially all species investigated. Often this was found to be due to large amounts of redundant sequences or absence of data from ESTs or partial gene prediction hits. After manual curation we assembled the PDZome database, which contains the best complete and non-redundant set of PDZ domains for the species listed to date. (B) Graph depicting both the number of PDZ domains and PDZ encoding genes found in the various genomes investigated. (C) Logarithmic plot of a subset of species from the PDZome dataset (Hs, Mm, Gg, Xt, Tr, Tn, Ci, Dm, Ce, At, Pa and Ec). This figure also shows the R-square and trend function obtained from the data. (D) Logarithmic plot for the data retrieved from the Superfamily database (http://supfam.mrc-lmb.cam.ac.uk/SUPERFAMILY/), using the similar subset of species as in Fig. S1C. (D) Logarithmic plot for the Pfam (http://pfam.sanger.ac.uk/) and SMART (http://smart.embl-heidelberg.de/smart/set_mode.cgi?NORMAL=1) databases as in Fig. S1C. (E) Logarithmic plot using the full set of species investigated for the PDZome dataset, as in Fig. 1C of the main text. The R-square value of this graph is inferior to the one in S1C, likely as a result of the inclusion of data from unfinished genome projects. (F) Distribution of the number of PDZs per gene per organism in percentages shows that PDZ domains are not evenly distributed over the genes and that multi-PDZ genes are underrepresented. The graph shows furthermore an increase in PDZ gene complexity during metazoan evolution and highly complex genes in Monosiga brevicollis, with up to 22 PDZs per gene. The gene complexity in the non-metazoan species investigated is low. (TIF) [file pone.0016047.s001.tif]

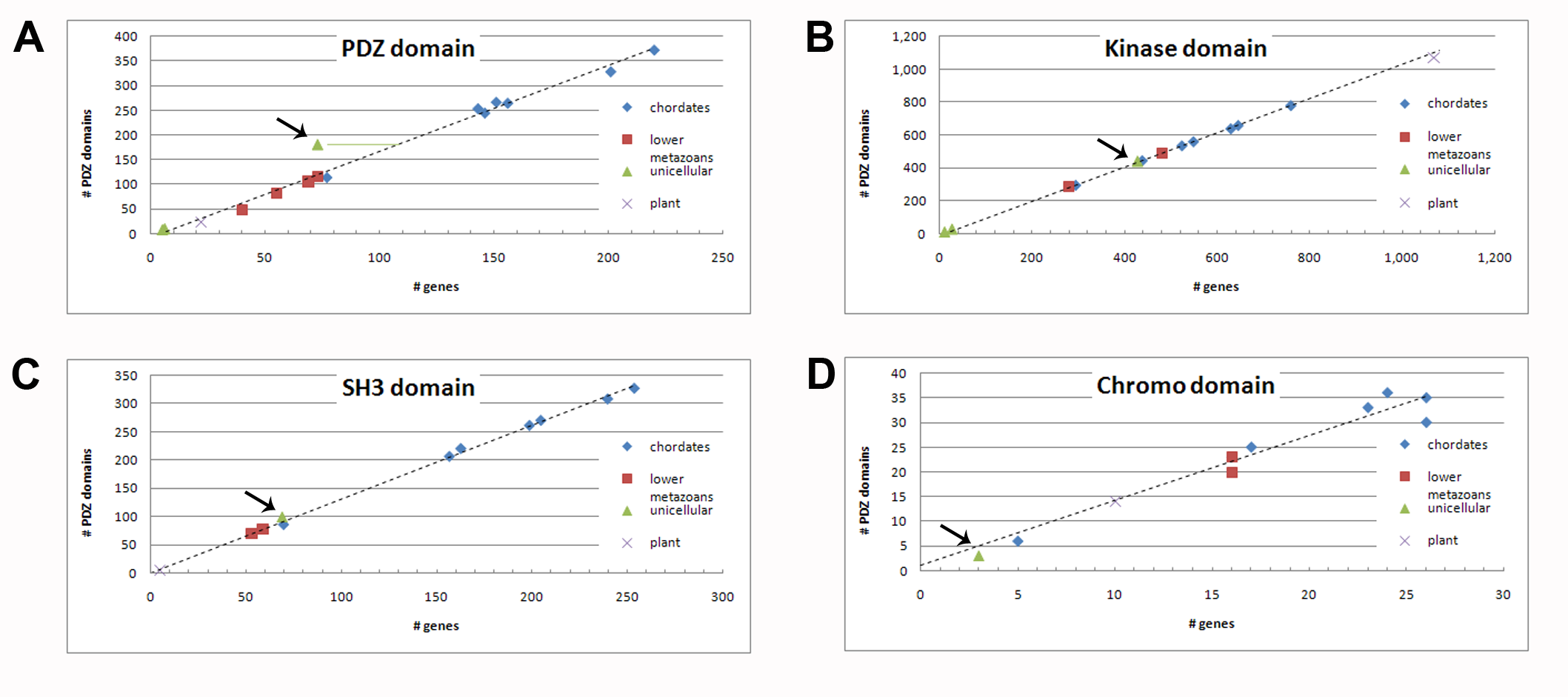

Supplement: Figure S2 — Relation between domain content and genome. (A) Correlation between the number of PDZs per genome and the number of PDZ encoding genes. Arrow indicates the Monosiga brevicollis data point. (B) Correlation between the number of kinase domains per genome and the number of kinase domain encoding genes. (C) Correlation between the number of SH3 domains per genome and the number of SH3 encoding genes. (D) Correlation between the number of chromo domains per genome and the number of chromo domain encoding genes. (TIF) [file pone.0016047.s002.tif]

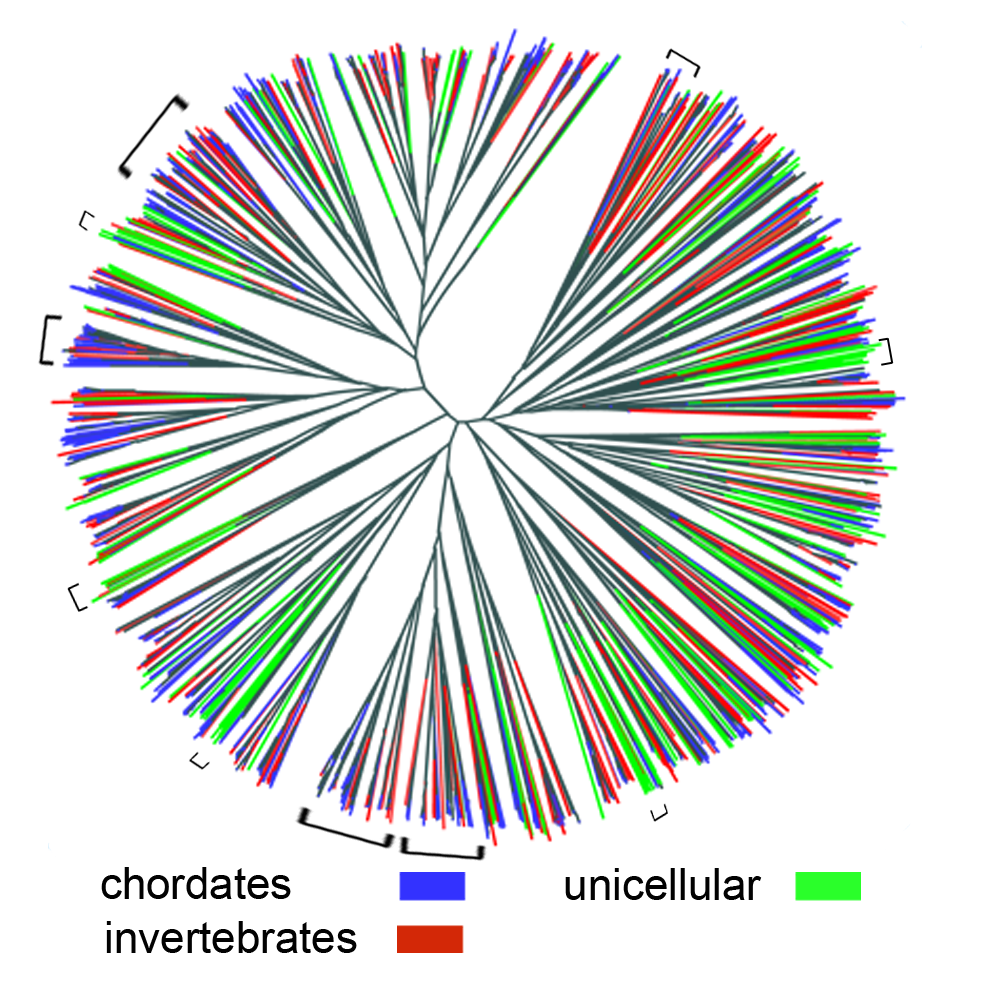

Supplement: Figure S3 — Clustering of PDZ sequences. Hierarchical clustering after multiple sequence alignment was color coded for chordate (blue), invertebrate (red) and unicellular (green) species. This illustrates that specific clusters of PDZ domains exist that are specific for unicellular or metazoan species (indicated with brackets). The latter are mostly composed of PDZ binding pocket sequences encoded by the Monosiga brevicollis genome, suggesting that these arose specifically in this species and that the Monosiga brevicollis PDZ domains were not transferred through horizontal gene transfer, as was proposed previously for unicellular organisms. (TIF) [file pone.0016047.s003.tif]

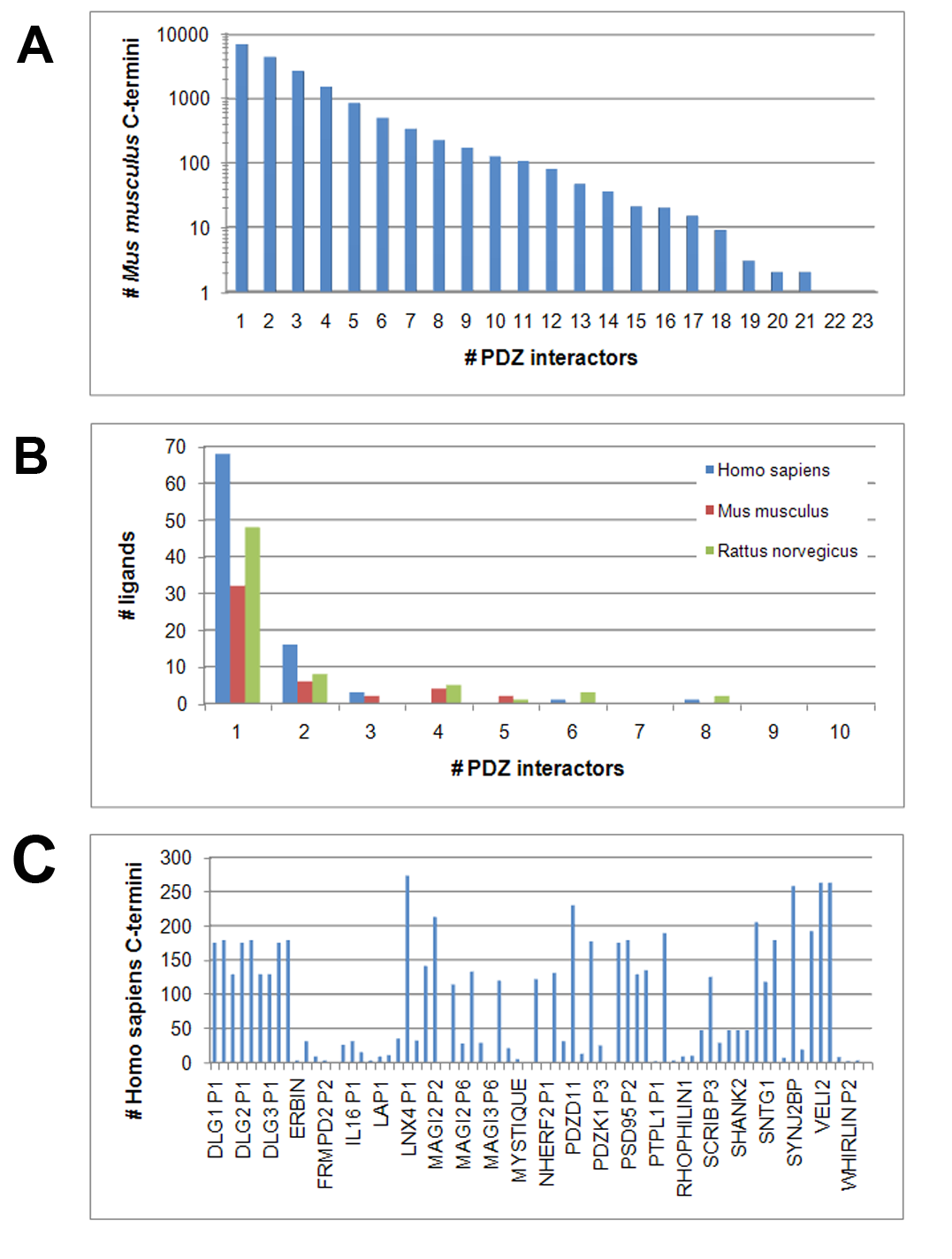

Supplement: Figure S4 — Extensive PDZ binding overlap in other organisms. (A) Mouse PDZ-ligand interaction predicted by Stiffler et al. for a redundant set of mouse proteins. (B) The observation that multiple human PDZ domains bind multiple ligands is also apparent from our analysis of an experiment-based set of interactions extracted from the PDZbase. (C) Number of interactions per PDZ as predicted with the set of 22,997 human C-termini from non-redundant (longest transcript) Ensembl protein sequences. (TIF) [file pone.0016047.s004.tif]

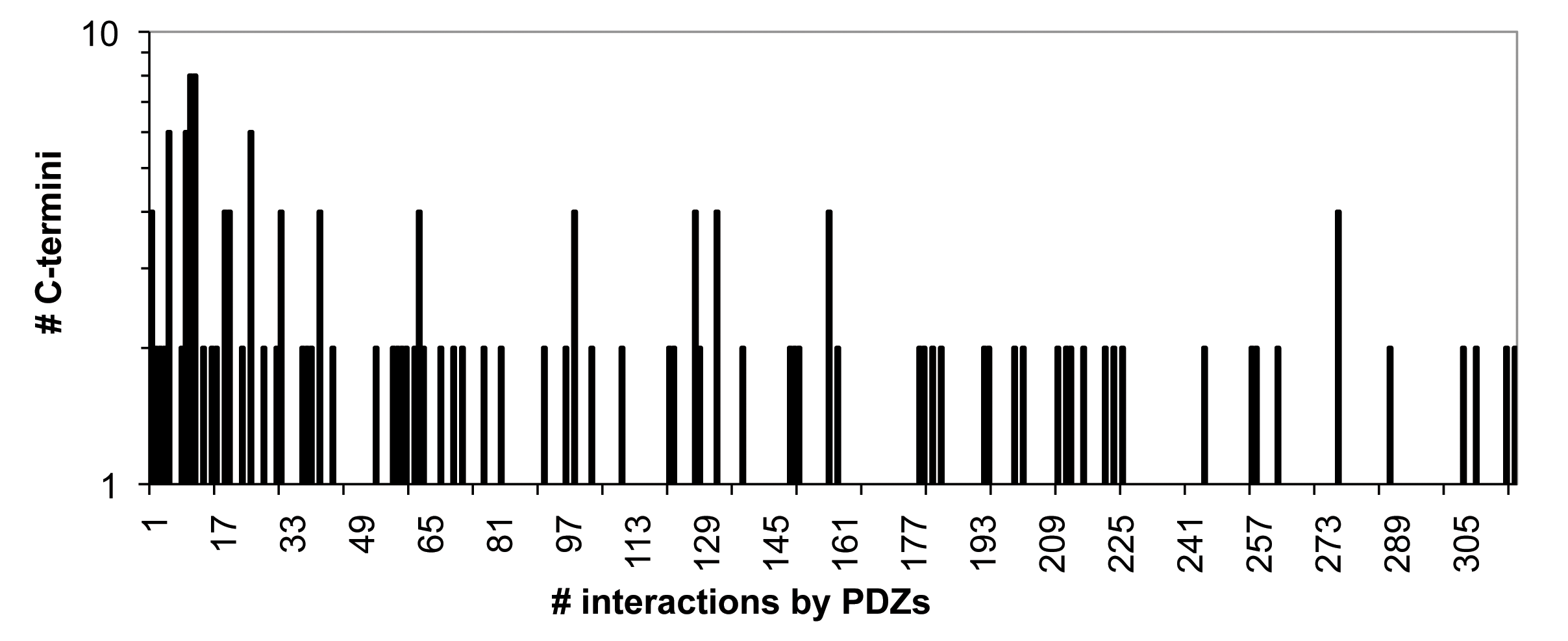

Supplement: Figure S5 — Genome-wide prediction of PDZ binding using an alternative algorithm. Extensive PDZ binding overlap in the human genome as predicted using the method from Hui and Bader. Compared to the results obtained with the method by Chen et al (Fig. 3A), a lot more overlap is seen, with many more C-termini being bound by different PDZs. (TIF) [file pone.0016047.s005.tif]

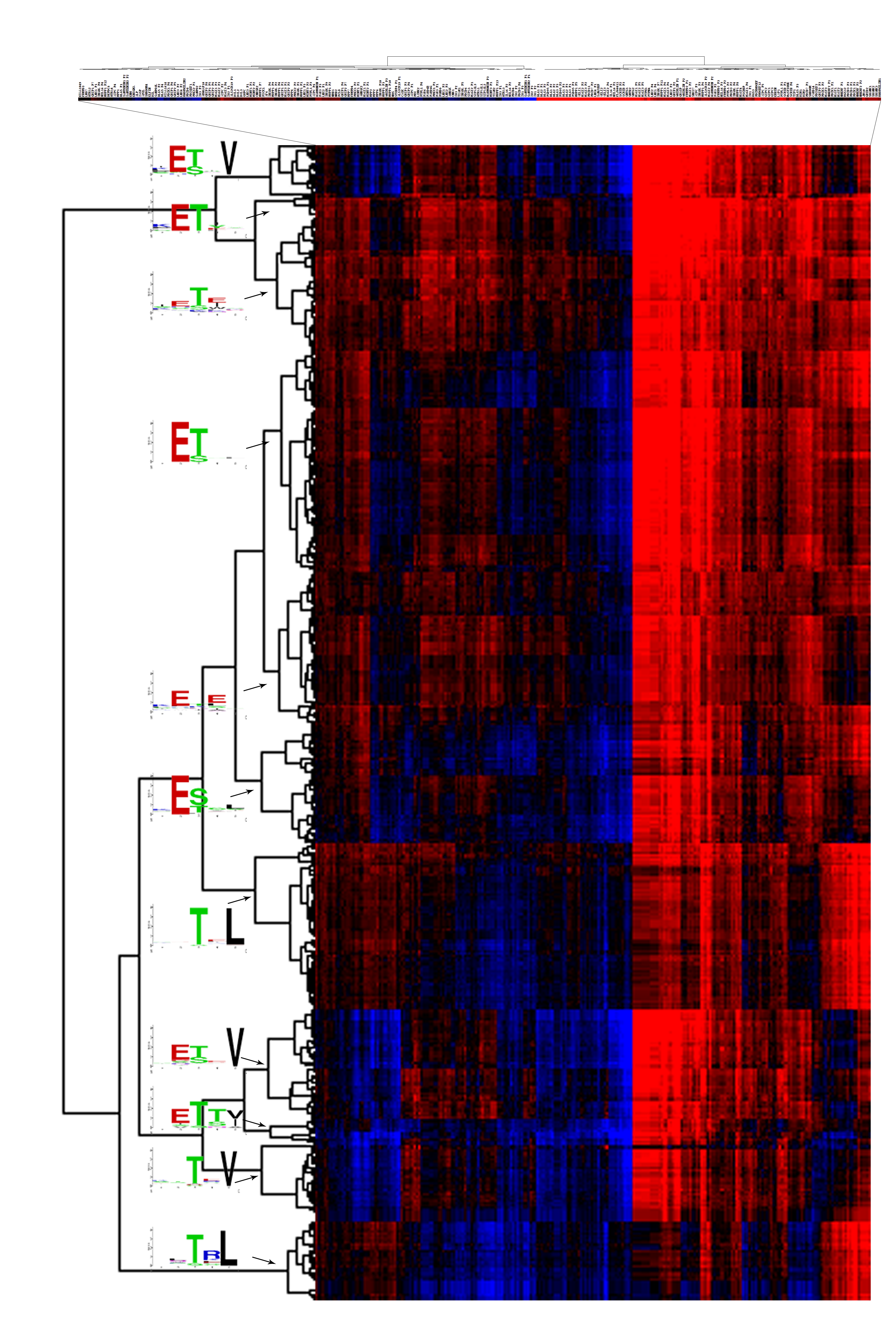

Supplement: Figure S6 — Clustering of PDZ domains and their ligands. Genome-wide hierarchical clustering was used to place human C-termini and PDZ domains with similar binding profiles in close proximity. Beside clusters of ligands on the left of the cluster graph (presented by their amino acid consensus), this heat map also reveals two main PDZ groups: a ligand specific group (marked ‘a’) with on average 1 ligand and a promiscuous group (marked ‘b’) with on average 55 ligands, both at a FPR of 6.27%. Positive psi scores are indicated in red and the negative scores are indicated in blue. (TIF) [file pone.0016047.s006.tif]
