# Supplementary material for: Genome-Wide Analysis of PDZ Domain Binding Reveals Inherent Functional Overlap within the PDZ Interaction Network
Source: PLoS One. 2011 Jan 24;6(1):e16047. doi: 10.1371/journal.pone.0016047 (PMC3026046; doi:10.1371/journal.pone.0016047)
Supplement: File S1 — Table showing number of essential genes encoding PDZ, SH3, Kinase or Chromo domains. (DOC) [file pone.0016047.s007.doc]

Supplemental file S2

|  | *Caenorhabditis elegans* | | | *Saccharomyces cerevisiae* | | | *Escherichia coli* | | |
| --- | --- | --- | --- | --- | --- | --- | --- | --- | --- |
|  | **genes** | **essential genes** | **% essential** | **genes** | **essential genes ^#^** | **% essential** | **genes** | **essential genes ^#^** | **% essential** |
| **PDZ** | 55* | 51 | 92.7 | 2 | 0 | 0 | 6 | 1 | 16.7 |
| **SH3** | 52 | 52 | 100.0 | 23 | 1 | 4.3 | none | - | - |
| **Kinase** | 480 | 471 | 98.1 | 132 | 19 | 14.4 | 10 | 1 | 10.0 |
| **Chromo** | 16 | 14 | 87.5 | 1 | 0 | 0 | none | - | - |

* Number contains newly, manually identified genes in the genome, which have not been annotated or investigated previously.

^#^ Using the definition that deletion of a gene results in a lethal phenotype under rich growth conditions.
