# Supplementary material for: Genome-Wide Analysis of PDZ Domain Binding Reveals Inherent Functional Overlap within the PDZ Interaction Network
Source: PLoS One. 2011 Jan 24;6(1):e16047. doi: 10.1371/journal.pone.0016047 (PMC3026046; doi:10.1371/journal.pone.0016047)
Supplement: File S3 — Supplemental text with more details on the PDZ dataset described in this study. (DOC) [file pone.0016047.s009.doc]

**Supplemental text**

***Data set***

In a systems biology approach towards the PDZ superfamily we have combined information from multiple databases and present here the manually curated PDZ domain complements (“PDZomes”) of several vertebrate and invertebrate species. PDZ domains are indentified by various databases (*e.g.*, SMART and Pfam; see Material Methods), but a caveat often associated with these automated compilations are a high degree of redundancy and the presence of (predicted) genes and various splice forms. These databases thus provide contradicting numbers for the total amount of protein domains per genome, including the PDZ domain (Fig. S1A).

There are various approaches to correct for this redundancy, such as 50% overlap scoring [1], or the correction that is implemented into the Superfamily database [2], which allows users to download sequences of the longest transcript only. Indeed, these approaches give significant improvements with respect to assessing the number of domains encoded per genome (see Fig. S1A for the latter). But careful cross-comparison of various protein and genome databases, reveals that even the picture obtained via this high-throughput way is not always complete (Fig. S1A). We therefore assembled a manually curated dataset (Fig. S1B, file S2), which represents PDZ data from six vertebrate genomes (*Homo sapiens*, *Mus musculus, Gallus gallus, Xenopus tropicalis*, *Takifugu rubripes* and *Tetraodon nigroviridis*), a lower chordate (sea squirt (*Ciona intestinalis*)), various lower metazoans (the purple sea urchin (*Strongylocentrotus purpuratus)*, the arthropods fruitfly (Drosophila melanogaster) and nematode (Caenorhabditis elegans), a cnidaria (*Hydra vulgaris*)), a choanoflagellate (*Monosiga brevicollis*), a plant (*Arabidopsis thaliana*), a fungus (*Saccharomyces cerevisiae*) and two bacterial genomes (*Escherichia coli* and *Pseudomonas auruginosa)*. Cross-verification among the various genomes and databases queried, suggests that our manually refined and verified dataset is both more complete and more accurate in its representation of the number of PDZ domains encoded per genome and per gene (Fig. S1A, S1C-E).

Logarithmic plotting of the various genome sizes and their associated PDZomes shows that this increase in the total number of PDZ domains encoded and the number of PDZ encoding genes can be approximated by a power-law fit ($N=aG^{b}$, where *N* is a number presenting a specific aspect of the PDZome content (either in domains or genes), *G* the genome size, *a* the off-set and *b* the power-law exponent). Using a similar subset of spieces in a logarithmic plot of the non-redundant Superfamily database entries (Fig. S1D), we show that this database gives a power-law function comparable to our manually curated dataset (Fig. S1C), yet still performs less as indicated by the inferior correlation coefficient (r^2^) value. The data retrieved from the SMART database, follows a power-law function that deviates due to a much smaller power-law exponent (Fig. S1E), visualizing therefore easily the specific overestimation of the domain content in the smaller genomes (bacterial) and an underestimation in the higher metazoans as seen in Fig. S1A. The data from the Pfam database could be fit with an almost equal power-law function compared our dataset (Fig. S1C, E), albeit with an R-square value of 0.36 as a result of numerous over- and underestimations (*e.g.*, caused by redundancy), as suggested by Fig. S1A as well. We conclude that our manually curated dataset is superior compared to all three public databases and should at this time thus give the best approximation of the human PDZ domain content (267 PDZs) for further analysis.

***PDZome expansion***

Previously, major differences were identified among protein domains with regard to their presence in the genome and their protein domain recombination promiscuity [3,4]. It was found that some domains are always high ranking (*e.g.*, SH3 and PH domains), whereas others are only present in relatively low numbers or merely display their promiscuity in higher organisms. To determine the relative evolutionary trend of the PDZ domain we compared it to the average superfamily size, two previously identified high ranking superfamilies and one relatively low ranking superfamily [3,4]. Judged against the SH3 domain - a widely conserved protein-protein interaction domain specifically involved in binding proline-rich regions [5] - the power-law fit to the PDZ domain data has a smaller exponent (Fig. S2C), which suggests that the evolutionary pressures on both domains were different. The kinase superfamily on the other hand compares more closely to the power-law behavior observed for the PDZ superfamily, differing in power-law exponent by only 0.02 (Fig. 1C, S2B). When we put these three evolutionary trends in the light of the overall protein (proteome) and domain content per genome we find that all three domains show steeper trends relative to the proteome (Fig. 1C), which underlines their fundamental evolutionary importance and abundance, and suggests that the three protein domains were evolutionary favored over other protein domains. A less abundant protein-protein interaction domain like the chromo domain - a protein-protein interaction domain involved in histone binding and with a SH3-like barrel fold [6] - fitted well to the a function similar to the average superfamily increase, suggesting consequently a similar evolutionary expansion (Fig. S2D).

Interestingly, the PDZ domain content and distribution in the choanoflagellate *Monosiga brevicollis* appeared markedly different from lower metazoans (Fig. 1A, 1B). A similar observation can be made for the SH3 and the kinase domain as well (Fig. S2), with the latter supported by recently published observations [7]. The chromo domain does not show this particular increase relative to other unicellular organisms and lower metazoans (Fig. S2), suggesting that like in metazoans, the highly promiscuous protein domains like PDZ, SH3 and kinase were positively selected for in this unicellular organism as well. Furthermore, it argues against previous claims about PDZ domain content expansion with multicellularity [8,9]. As a likely result of the partial genome duplications in teleost fish [10], numbers for *Takifugu rubripes* (373 PDZ domains) and *Tetraodon nigroviridis* (329 PDZ domains) are higher.

**References**

1. Karev G, Wolf Y, Rzhetsky A, Berezovskaya F, EV K (2002) Birth and death of protein domains: a simple model of evolution explains power law behavior. BMC Evol Biol 2: 18.

2. Gough J, Chothia C (2002) SUPERFAMILY: HMMs representing all proteins of known structure. SCOP sequence searches, alignments and genome assignments. Nucleic Acids Res 30: 268-272.

3. Basu MK, Carmel L, Rogozin IB, Koonin EV (2008) Evolution of protein domain promiscuity in eukaryotes. Genome Res 18: 449-461.

4. Wuchty S, Almaas E (2005) Evolutionary cores of domain co-occurrence networks. BMC Evol Biol 5: 24.

5. Kami K, Takeya R, Sumimoto H, Kohda D (2001) Diverse recognition of non-PxxP peptide ligands by the SH3 domains from p67(phox), Grb2 and Pex13p. EMBO J 21: 4268-4276.

6. Jacobs S, Taverna S, Zhang Y, Briggs S, Li J, et al. (2001) Specificity of the HP1 chromo domain for the methylated N-terminus of histone H3. EMBO J 20: 5232-5241.

7. Manning G, Young S, Miller W, Zhai Y (2008) The protist, Monosiga brevicollis, has a tyrosine kinase signaling network more elaborate and diverse than found in any known metazoan. Proc Natl Acad Sci U S A 105: 9674-9679.

8. Ponting CP, Pallen MJ (1999) beta-propeller repeats and a PDZ domain in the tricorn protease: predicted self-compartmentalisation and C-terminal polypeptide-binding strategies of substrate selection. FEMS Microbiology Letters 179: 447-451.

9. Pallen MJ, Ponting CP (1997) PDZ domains in bacterial proteins. Molecular Microbiology 26: 411-413.

10. Volff JN (2004) Genome evolution and biodiversity in teleost fish. Heredity 94: 280-294.
