# Supplementary material for: Genome-Wide Analysis of PDZ Domain Binding Reveals Inherent Functional Overlap within the PDZ Interaction Network
Source: PLoS One. 2011 Jan 24;6(1):e16047. doi: 10.1371/journal.pone.0016047 (PMC3026046; doi:10.1371/journal.pone.0016047)
Supplement: File S5 — Table showing a PMW comparison of the two computational methods used in this study. (DOC) [file pone.0016047.s011.doc]

| **PDZ** | **Chen et al method** | **Hui and Bader method** |
| --- | --- | --- |
| ***CARD14-P1*** |  |  |
| ***DLG1-P1*** |  |  |
| ***DLG1-P2*** |  |  |
| ***DLG1-P3*** |  |  |
| ***DLG5-P3*** |  |  |
| ***DVL1-P1*** |  |  |
| ***DVL2-P1*** |  |  |
| ***DVL3-P1*** |  |  |
| ***ERBIN-P1*** |  |  |
| ***GIPC1-P1*** |  |  |
| ***GRID2-P1*** |  |  |
| ***GRIP1-P2*** |  |  |
| ***GRIP2-P3*** |  |  |
| ***INADL-P2*** |  |  |
| ***INADL-P3*** |  |  |
| ***INADL-P6*** |  |  |
| ***INADL-P7*** |  |  |
| ***MAGI1-P2*** |  |  |
| ***MAGI1-P4*** |  |  |
| ***MAGI1-P5*** |  |  |
| ***MAGI1-P6*** |  |  |
| ***MAST1-P1*** |  |  |
| ***MAST2-P1*** |  |  |
| ***MAST3-P1*** |  |  |
| ***MUPP1-P4*** |  |  |
| ***MUPP1-P5*** |  |  |
| ***MUPP1-P6*** |  |  |
| ***MUPP1-P8*** |  |  |
| ***MUPP1-P9*** |  |  |
| ***MUPP1-P11*** |  |  |
| ***LAP1-P1*** |  |  |
| ***LNX2-P1*** |  |  |
| ***LNX2-P3*** |  |  |
| ***LNX2-P4*** |  |  |
| ***MYSTIQUE-P1*** |  |  |
| ***NHERF1-P1*** |  |  |
| ***NHERF1-P2*** |  |  |
| ***NHERF2-P1*** |  |  |
| ***NHERF2-P2*** |  |  |
| ***RIL-P1*** |  |  |
| ***RIM1-P1*** |  |  |
| ***RIM2-P1*** |  |  |
| ***SCRIB1-P1*** |  |  |
| ***SCRIB1-P2*** |  |  |
| ***SCRIB1-P3*** |  |  |
| ***SCRIB1-P4*** |  |  |
| ***SHANK1-P1*** |  |  |
| ***SHANK2-P1*** |  |  |
| ***SNTB2-P1*** |  |  |
| ***SNTG1-P1*** |  |  |
| ***SNTG2-P1*** |  |  |
| ***WHIRLIN1-P1*** |  |  |
| ***WHIRLIN2-P1*** |  |  |
| ***ZO1-P1*** |  |  |
