# Supplementary material for: Genome-Wide Analysis of PDZ Domain Binding Reveals Inherent Functional Overlap within the PDZ Interaction Network
Source: PLoS One. 2011 Jan 24;6(1):e16047. doi: 10.1371/journal.pone.0016047 (PMC3026046; doi:10.1371/journal.pone.0016047)
Supplement: File S6 — Table listing the amino acid sequences of the PDZ domains and their mutants that were used for binding analysis. (DOC) [file pone.0016047.s012.doc]

## The PDZ domains cloned as cDNA sequences into the pGEX vector correspond to the amino acid sequences listed below; postions of site directed mutagenesis are indicated in bold and capital letters. Corresponding residues in the wild type domain are indicated as well for comparison.

| LMO7 | TPGKSLDFGFTIKWDIPGIFVASVEAGSPAEFSQLQVDDEIIAINNTKFSYN  D**S**KEW**E**EAMAKQETGHLVMDVRRYGKAGSP |
| --- | --- |
| LMO7 mutant | TPGKSLDFGFTIKWDIPGIFVASVEAGSPAEFSQLQVDDEIIAINNTKFSYN  D**H**KEW**V**EAMAKQETGHLVMDVRRYGKAGSP |
| SHANK1 | EGFGFVLRGAKAQTPIEEFTPTPAFPALQYLESVDEGGVAWRAGLRMGDF  LIEVNGQNVKVG**H**RQV**V**NMIRQGGNTLMVKVVMVTRHPD |
| SHANK1 mutant | EGFGFVLRGAKAQTPIEEFTPTPAFPALQYLESVDEGGVAWRAGLRMGDF  LIEVNGQNVKVG**S**RQV**E**NMIRQGGNTLMVKVVMVTRHPD |
| ZO1PDZ1 | HRAPGFGFGIAISGGRDNPHFQSGETSIVISDVLKGGPAEGQLQENDR  VAMVNGVSMDNVE**H**AFA**V**QQLRKSGKNAKITIRRKKKVQIP |
| ZO1PDZ1 mutant | HRAPGFGFGIAISGGRDNPHFQSGETSIVISDVLKGGPAEGQLQENDR  VAMVNGVSMDNVE**L**AFA**K**QQLRKSGKNAKITIRRKKKVQIP |
| ZO1PDZ2 | KVTLVKSRKNEEYGLRLASHIFVKEISQDSLAARDGNIQEGDVVLKI  NGTVTENMS**L**TDA**K**TLIERSKGKLKMVVQRDEERATLLNVPD |
| ZO1PDZ2 mutant | KVTLVKSRKNEEYGLRLASHIFVKEISQDSLAARDGNIQEGDVVLKI  NGTVTENMS**H**TDA**V**TLIERSKGKLKMVVQRDEERATLLNVPD |
