# Supplementary material for: Genome-Wide Analysis of PDZ Domain Binding Reveals Inherent Functional Overlap within the PDZ Interaction Network
Source: PLoS One. 2011 Jan 24;6(1):e16047. doi: 10.1371/journal.pone.0016047 (PMC3026046; doi:10.1371/journal.pone.0016047)
Supplement: File S7 — Table listing the peptide sequences that were used in the binding analysis. (DOC) [file pone.0016047.s013.doc]

| **Ensembl number of C-terminal sequence** | **Peptide sequence** |
| --- | --- |
| ENSP00000282018 | NNGSVWLRKETRV |
| ENSP00000295598 | NNGGGWVEKETYY |
| ENSP00000309968 | NNGNRVDSKETEC |
| ENSP00000351197 | NNGGGHSGSETSL |
| ENSP00000285083 | NNGKEHICFEKWW |
| ENSP00000263284 | NNGEYMNRLDMRS |
| ENSP00000319705 | NNGPDLKCITTNL |
| ENSP00000279396 | NNGRESHWSRTRL |
| ENSP00000282441 | NNGLDKESFLTWL |
| ENSP00000003084 | NNGTEEEVQDTRL |
| ENSP00000353036 | NNGAVGVGAILWL |
| ENSP00000248150 | NNGWVEKGKCTIL |
| ENSP00000345468 | NNGQGRWDHETIV |
| ENSP00000299339 | NNGSKQFDKNAYV |
| ENSP00000328326 | NNGQERLEYETTL |
| ENSP00000282018 | NNGSVWLRKETRV |
| ENSP00000351197 | NNGGGHSGSETSL |
| ENSP00000326432 | NNGSRSSSVDYIL |
