# Supplementary material for: Genome-Wide Analysis of PDZ Domain Binding Reveals Inherent Functional Overlap within the PDZ Interaction Network
Source: PLoS One. 2011 Jan 24;6(1):e16047. doi: 10.1371/journal.pone.0016047 (PMC3026046; doi:10.1371/journal.pone.0016047)
Supplement: File S8 — Raw interaction data between PDZ and peptide ligands. (DOC) [file pone.0016047.s014.doc]

| ***ensembl nr*** | ***peptide sequence*** | ***psi score***  ***(predicted)*** | ***Average (n=6)***  ***(450 nm extinction)*** | ***SD*** |
| --- | --- | --- | --- | --- |
|  |  | **GST Control** |  |  |
| *n.a.* | *n.a* | 0 | 0.461333333 | 0.02966929 |
|  |  |  |  |  |
|  |  | **LMO7 wt** |  |  |
| ENSP00000282018 | NNGSVWLRKETRV | 0.139611 | 0.443833333 | 0.03202811 |
| ENSP00000295598 | NNGGGWVEKETYY | 0.187028 | 0.459 | 0.0367913 |
| ENSP00000309968 | NNGNRVDSKETEC | 0.324775 | 0.480166667 | 0.07067225 |
| ENSP00000351197 | NNGGGHSGSETSL | 0.11664 | 0.492 | 0.07368311 |
| ENSP00000263284 | NNGEYMNRLDMRS | 0.000204 | 0.468166667 | 0.04452153 |
|  |  |  |  |  |
|  |  | **LMO7 mutant** | |  |
| ENSP00000282018 | NNGSVWLRKETRV | 0.68371 | 3.109666667 | 0.11100225 |
| ENSP00000295598 | NNGGGWVEKETYY | 0.730103 | 3.034333333 | 0.12646844 |
| ENSP00000309968 | NNGNRVDSKETEC | 0.826118 | 3.011333333 | 0.07029546 |
| ENSP00000351197 | NNGGGHSGSETSL | 0.645571 | 2.341333333 | 0.225719 |
| ENSP00000263284 | NNGEYMNRLDMRS | 0.030904 | 0.499666667 | 0.01399524 |
|  |  |  |  |  |
|  |  | **ZO1 P2 wt** |  |  |
| ENSP00000319705 | NNGPDLKCITTNL | 0.148812 | 0.462666667 | 0.04774376 |
| ENSP00000279396 | NNGRESHWSRTRL | 0.071939 | 0.456666667 | 0.03656592 |
| ENSP00000282441 | NNGLDKESFLTWL | 0.17944 | 0.482716667 | 0.06561373 |
| ENSP00000003084 | NNGTEEEVQDTRL | 0.062795 | 0.476666667 | 0.020221 |
| ENSP00000353036 | NNGAVGVGAILWL | 0.280826 | 0.540333333 | 0.08495332 |
|  |  |  |  |  |
|  |  | **ZO1 P2 mutant** | |  |
| ENSP00000319705 | NNGPDLKCITTNL | 0.570652 | 3.016333333 | 0.08431528 |
| ENSP00000279396 | NNGRESHWSRTRL | 0.4996 | 1.706333333 | 0.14728295 |
| ENSP00000282441 | NNGLDKESFLTWL | 0.498745 | 2.926166667 | 0.1484108 |
| ENSP00000003084 | NNGTEEEVQDTRL | 0.457267 | 2.191166667 | 0.23873116 |
| ENSP00000353036 | NNGAVGVGAILWL | 0.272287 | 0.5035 | 0.02511374 |
|  |  |  |  |  |
|  |  | **ZO1 P1 wt** |  |  |
| ENSP00000295598 | NNGGGWVEKETYY | 0.758104 | 2.9725 | 0.10506522 |
| ENSP00000309968 | NNGNRVDSKETEC | 0.562245 | 1.976666667 | 0.07774488 |
| ENSP00000248150 | NNGWVEKGKCTIL | 0.557715 | 0.527166667 | 0.01851936 |
| ENSP00000345468 | NNGQGRWDHETIV | 0.636677 | 3.039333333 | 0.1306517 |
| ENSP00000299339 | NNGSKQFDKNAYV | 0.865819 | 3.006 | 0.10014789 |
|  |  |  |  |  |
|  |  | **ZO1 P1 mutant** | |  |
| ENSP00000295598 | NNGGGWVEKETYY | 0.25249 | 0.517666667 | 0.04461689 |
| ENSP00000309968 | NNGNRVDSKETEC | 0.09192 | 0.510666667 | 0.01697842 |
| ENSP00000248150 | NNGWVEKGKCTIL | 0.119993 | 0.485333333 | 0.03138577 |
| ENSP00000345468 | NNGQGRWDHETIV | 0.188507 | 0.525833333 | 0.03116034 |
| ENSP00000299339 | NNGSKQFDKNAYV | 0.777431 | 2.992166667 | 0.0955226 |
|  |  |  |  |  |
|  |  | **SHANK1 wt** |  |  |
| ENSP00000328326 | NNGQERLEYETTL | 0.75674 | 2.775 | 0.11632025 |
| ENSP00000282018 | NNGSVWLRKETRV | 0.665923 | 3.035666667 | 0.0542611 |
| ENSP00000295598 | NNGGGWVEKETYY | 0.616047 | 2.8285 | 0.10759879 |
| ENSP00000351197 | NNGGGHSGSETSL | 0.960768 | 2.797538286 | 0.06878856 |
| ENSP00000326432 | NNGSRSSSVDYIL | 0.504731 | 0.506 | 0.04116795 |
|  |  |  |  |  |
|  |  | **SHANK1 mutant** | |  |
| ENSP00000328326 | NNGQERLEYETTL | 0.295234 | 0.480333333 | 0.03402744 |
| ENSP00000282018 | NNGSVWLRKETRV | 0.121824 | 0.496 | 0.01909974 |
| ENSP00000295598 | NNGGGWVEKETYY | 0.072972 | 0.482 | 0.0321123 |
| ENSP00000351197 | NNGGGHSGSETSL | 0.431837 | 0.482833333 | 0.02596472 |
| ENSP00000326432 | NNGSRSSSVDYIL | 0.491398 | 0.47 | 0.02841654 |
